# Maternal consumption of canola oil suppressed mammary gland tumorigenesis in C3(1) TAg mice offspring

**DOI:** 10.1186/1471-2407-10-81

**Published:** 2010-03-06

**Authors:** Gabriela Ion, Juliana A Akinsete, W Elaine Hardman

**Affiliations:** 1 Department of Biochemistry and Microbiology, Marshall University School of Medicine, Huntington, West Virginia, USA

## Abstract

**Background:**

Maternal consumption of a diet high in omega 6 polyunsaturated fats (n-6 PUFA) has been shown to increase risk whereas a diet high in omega 3 polyunsaturated fats (n-3 PUFA) from fish oil has been shown to decrease risk for mammary gland cancer in female offspring of rats. The aim of this study was to determine whether increasing n-3 PUFA and reducing n-6 PUFA by using canola oil instead of corn oil in the maternal diet might reduce the risk for breast cancer in female offspring.

**Methods:**

Female SV 129 mice were divided into two groups and placed on diets containing either 10% w/w corn oil (which is 50% n-6 PUFA, control diet) or 10% w/w canola oil (which is 20% n-6 PUFA, 10% n-3 PUFA, test diet). After two weeks on the diets the females were bred with homozygous C3(1) TAg transgenic mice. Mother mice consumed the assigned diet throughout gestation and nursing of the offspring. After weaning, all female offspring were maintained on the control diet.

**Results:**

Compared to offspring of mothers fed the corn oil diet (CO/CO group), offspring of mothers fed the canola oil diet (CA/CO group) had significantly fewer mammary glands with tumors throughout the experiment. At 130 days of age, the CA/CO group had significantly fewer tumors per mouse (multiplicity); the tumor incidence (fraction of mice with any tumor) and the total tumor weight (per mouse that developed tumor) was less than one half that of the CO/CO group. At 170 days of age, the total tumor weight per mouse was significantly less in the CA/CO group and if a tumor developed the rate of tumor growth rate was half that of CO/CO group. These results indicate that maternal consumption of canola oil was associated with delayed appearance of mammary gland tumors and slowed growth of the tumors that developed.

**Conclusions:**

Substituting canola oil for corn oil is an easy dietary change for people to make; such a change to the maternal diet may decrease risk for breast cancer in the daughter.

## Background

It has been shown that diets that contain high amounts of omega 6 polyunsaturated fatty acids (PUFA) increase the growth rates of cancers [1] whereas omega 3 PUFA have been shown to reduce cancer growth rates [2] and have been suggested as cancer preventive agents [3]. The type of fat consumed by the mother during pregnancy and nursing of the offspring has also been shown to influence mammary gland cancer risk in the offspring. A maternal diet that contained a high (versus low) amount of omega 6 fatty acids increased the risk for mammary gland cancer in the carcinogen treated offspring [4]. Compared to a corn oil diet, maternal consumption of either an olive oil containing diet (high in omega 9 fatty acid) [5] or of a diet containing long chain omega 3 PUFA from fish oil [6] has been shown to decrease carcinogen induced mammary gland cancer in rat offspring. We have reported that the 18 carbon omega 3 PUFA found in canola oil also effectively slowed the growth of implanted mammary gland cancers [7]. We hypothesized that a maternal diet that contained canola oil instead of corn oil would increase maternal dietary omega 3 PUFA and thus reduce the risk for mammary gland cancer in the offspring.

The C3(1) SV40 T-antigen transgenic mouse was selected as the model for this study. This mouse is a well characterized model developed in the laboratory of Dr. Jeffrey E. Green jeffrey.green1@nih.gov. The transgene uses the 5' flanking region of the rat C3(1) prostate steroid binding protein to target expression of the SV 40 large T-antigen in the mammary gland and prostate [8]. The female mice develop invasive mammary gland tumors by 16 weeks of age [8]. The tumors are similar to human infiltrating ductal carcinomas, are hormone responsive at early stages but become hormone independent in later stages [8] similar to advanced human breast cancers. The lesions develop in a predictable time, thus, this mouse is a good model to study alterations in tumorigenesis and progression [8]. The T-antigen is thought to induce cancer by inactivation of p53 [9] and Rb [10], two proteins that are involved in cell cycle regulation. Both p53 and Rb are tumor suppressors and are frequently mutated in human breast cancer. Since the mouse has a strong transgenic tumor promoter, complete prevention of tumors would not be expected but a delay in tumor development or slower tumor growth compared to a control group would indicate cancer preventive benefit.

Most studies that have tested effects of omega 3 fatty acids on cancer have used the long chain omega 3 fatty acids, eicosapentaenoic (EPA, 20 carbons, 5 unsaturations) or docosahexaenoic (DHA, 22 carbons, 6 unsaturations) as the test fatty acids [examples: [11-15]. These fatty acids are most commonly found in fish, thus fish oil or fish oil concentrates are frequently incorporated into diets. However, the double bond in the omega 3 position is produced by plants not by animals, and can be found in plant products as the 18 carbon fat, α-linolenic acid (ALA). Good dietary sources of ALA include canola oil, walnuts and 'omega 3 eggs' [16]. Flaxseed or flaxseed oil is also a good source of ALA [17] but is often consumed as a dietary supplement rather than a part of the regular diet. Canola oil contains about 10% ALA and about 20% linoleic acid (LA), an 18 carbon omega 6 fatty acid [18], whereas corn oil contains about 50% LA and about 1% ALA [18]. A ratio of omega 3 to omega 6 fatty acids of somewhere between 1:1 and 1:4 has been suggested to be much healthier than the 1:10 to 1:25 ratio of omega 3 to omega 6 fatty acids contained in the usual Western diet [19].

The ALA that is consumed by animals (whether fish, mouse or human) may be metabolized without change or it may be elongated and desaturated to longer chain lipids of the omega 3 series [19]. Humans elongate and desaturate ALA, however there is controversy about the efficiency of this conversion [19]. Recent reports indicate that humans do convert ALA to measurable amounts of EPA and docosapentaenoic acid (22 carbons, 5 unsaturations, omega 3, DPA) and that the conversion is adequate to reduce measures of inflammation, indicating biologic activity of the omega 3 fatty acids in humans [20,21]. Long chain omega 3 PUFA incorporated in tissues have been shown to slow cancer growth in animal models by multiple mechanisms including: slowing proliferation, increasing apoptosis [22], increasing lipid peroxidation [15] and increasing oxidative damage in cancer cells but not normal cells [15]. Induction of these mechanisms is correlated with an increased amount of the n-3 PUFA in the tissues.

Johnson, et. al. used data from the National Health and Nutrition Survey, 1999-2002, to estimate the actual consumption of ALA and LA in the average American diet [23]. They report that the usual diet of adult Americans contains about 14.7 g of LA and about 1.5 g of ALA per day [23], almost 10 times as much LA as ALA. They estimated that if Americans replaced vegetable oils, butter, and margarine with canola oil or canola oil based margarine the intakes of ALA would increase to 2.6 g per day and of LA would decrease to 8.1 g per day [23]. This would change the ratio of these omega 3 and omega 6 fatty acids in the diet to about 1:3, a much healthier ratio that would better meet current dietary fat recommendations [23].

We have shown that the use of canola oil as a source of omega 3 PUFA significantly slowed the growth rate of MDA-MB 231 human breast cancers implanted in nude mice and that the decreased tumor growth rate was associated with increased long chain omega 3 PUFA in mouse tissues [7]. There is a suggestion that canola oil use may be beneficial against cancer in humans in that women who used canola oil for cooking had a lower risk for breast cancer than those who used hydrogenated fats or corn oil [24]. The use of canola oil instead of corn oil is a diet change that would be easy to make since canola oil could be substituted for corn oil in baking, frying and salad dressings. Our results indicate that such a dietary change could result in important health benefits, perhaps decreasing risk for cancer in the next generation.

## Methods

### Animals

Twenty, female SV129 mice, 6 weeks old were obtained from Charles River Laboratories (Wilmington, MA). Breeding pairs of mice bearing a transgene for the SV40 large T antigen with a C3(1) rat prostate steroid binding protein promoter were obtained from the Dr. Jeffrey Green. The female transgenic mice are expected to develop mammary gland cancer due to expression of the large T antigen in the mammary gland [25]. The transgenic line is maintained in the laboratory and all mice are genotyped to ensure presence of the transgene. All animal work was approved by the Marshall University School of Medicine Institutional Animal Care and Use Committee.

### Study design

Mice were quarantined for 2 weeks, and then moved to a study room. SV129 females were split into 2 groups and numbered for identification. Ten female mice were placed on a diet containing 10% w/w corn oil (control diet, see below) and ten female mice were placed on a diet containing 10% w/w canola oil (test diet). After 2 weeks these females were bred with homozygous C3(1)/TAg male mice. The hemizygous female pups from these breedings were the experimental mice NOT the wild type mother mice. Pups were weaned at 21 days old and placed on the corn oil containing diet, generating two experimental groups: corn/corn (CO/CO) and canola/corn (CA/CO) (the first diet is the maternal diet, the second diet is the pup's diet). Only the maternal diet of the CA/CO group contained canola oil not the diet of the experimental pups. The offspring were housed 3 to 4 in a cage, individually numbered for identification, and weighed weekly.

### Diet

Diets were prepared in the Marshall University School of Medicine animal diet prep room. Diet composition is shown in Table 1 and was formulated to be isocaloric, isonutrient and more relevant to human consumption than the high fat diets used in many studies. (If a Western diet contains about 14.7 g of LA/day [23] the calories from LA are 6.6% of a 2000 calorie diet. The 10% corn oil mouse diet contained 10.9% of calories from LA.) The AIN-76A diet is adequate for the nutritional support of the mice [26]. The dry ingredients of the diet, except sugar, were obtained in bulk from MP Biomedicals (Solon, Ohio), sugar and oil were purchased locally (100% canola oil or 100% corn oil, no additives or preservatives). Batches of diet were prepared as needed, about each two weeks. The diet mixture was pressed into trays and cut into small squares. Individual cage sized portions (25-30 g) were stored in sealed containers at -20°C to prevent oxidation of the fat and bacterial growth in the food. Mice had free access to food and water and were fed fresh food 5 days per week. Food removed from cages was discarded.

**Table 1:** Composition of the diets

Ingredient	% of wt	Amount/100 g	Calories/100 g
Casein (protein)	20%	20 g	80

Sucrose	45%	45 g	180

Corn starch (carbohydrate)	15%	15 g	60

Alphacel (fiber)	5%	5 g	0

Choline bitartrate	0.2%	0.2 g	0

DL-methionine	0.3%	0.3 g	0

Mineral mix	3.5%	3.5 g	0

Vitamin mix	1.0%	1 g	0

Oil (fat) either corn oil or canola oil	10%	10 g	90

Total	100%	100 g	410

Total fat		90 g	90

Total protein		80 g	80

Total carbohydrate		60 g	240

The AIN-76A was slightly modified to contain 10% w/w corn oil (control diet) or 10% w/w canola oil (test diet). Control and canola diets were balanced for calories, nutrients, protein, fat and carbohydrate. The AIN-76A diet is adequate for growth and nutrition of mice.

### Transgene copy number

Real time PCR was used to verify the presence of the transgene in all experimental pups. Ear punches (two 2 mm punches, stored at -20°C until processing) were digested in digestion buffer [50 mM KCl, 1.5 mM MgCl_2_, 10 mm Tris pH 8.5, 0.01% Gelatin, 0.45% NP-40, 0.45% Tween 20 containing 140 mg/ml proteinase K(Shelton Scientific, Shelton, CN)], followed by dilution of the samples 1:40 in milliQ water (Milli-Q Advantage, Millipore, Massachusettes). Primers for the transgene (SV40 foward: ATA TGC CTT CAT CAG AGG AAT ATT C; SV40 reverse: TAA AGT TTT AAA CAG AGA GGA ATC TTT GC) and the VIC labeled SV40PROBE (VICCCC AGG CAC TCC TTT CAA GAC CTA GAA GGMGBNFQ) were purchased from Applied Biosystems (Foster City, CA). Beta-actin primers and FAM labeled probe (for an internal control) and PCR Master Mix were also purchased from Applied Biosystems. The rtPCR assay was performed according to the Applied Biosystems instructions on an ABI Prism 7000 (Applied Biosystems, Foster City, CA) instrument.

### Tumor growth rates, incidence, multiplicity and weight

Mice were palpated for tumors 3 times weekly from 90 days of age. Lengths and widths of palpable tumors were measured from the time of detection until euthanasia to estimate tumor volumes. Tumor volume was estimated using the formula: (Length × Width × Width)/2. Prism^© ^software (Graphpad, Inc., La Jolla, CA) was used to plot tumor growth curves and for regression analyses to determine the growth rate of each palpable tumor. A T-test was then used to compare the mean tumor growth rates between groups of mice.

Total tumor incidence, multiplicity and weights were determined at necropsy. The differences between groups and across time were statistically analyzed by two way analyses of variance, T-test, Fisher exact test or Mann-Whitney test as appropriate using Prism^© ^software.

### Necropsy

Mice were euthanized at 21, 110, 130, 150 and 170 days of age. Twenty one days of age was the time of weaning. The earliest time for tumors was expected to be 110 days of age, mice were euthanized each 20 days thereafter to follow the increase in tumor incidence and multiplicity. The left 4^th ^mammary gland was quickly removed and frozen in liquid nitrogen. All ten mammary glands were examined for the presence of a tumor 1 mm or larger. All tumors detected were measured, removed and weighed, thus total tumor weight and numbers includes many tumors that were too small to be detected by palpation. If tumor was large enough for further assay, it was flash frozen in liquid nitrogen. The number of tumors in each gland and the number of glands with tumor were recorded for every mouse. Samples of inguinal fat and liver were removed and frozen in liquid nitrogen until further analyses.

### Body weights

Body weights were measured each week and terminally. Statistical differences in mean body weight change between groups were determined using a T-test and Prism^© ^(Graphpad, Inc) software.

### Gas chromatography

The fatty acid compositions of mammary glands and liver at 3 weeks of age and 130 days of age were analyzed by gas chromatography. Frozen tissues were thawed and homogenized in distilled water containing 0.1% BHT to prevent oxidation of the fatty acids. Lipids were extracted with chloroform/methanol, the fatty acids were methylated followed by separation and identification using gas chromatography, as previously described [7]. Gas chromatography was done using a PerkinElmer Clarus 500 Gas Chromatograph (Shelton, CT) with a Elite-5 (5% Diphenyl) Dimethyl-polisiloxane Series Capillary Column (Length: 30 m, Inner Diameter: 0.25 mm), under the following conditions: initial temperature 150°C, ramp 1 at 175°C for 15 min, ramp 2 at 225°C for 50 min, ramp 3 at 250°C for 10 min, helium carrier gas flow rate of 1.60 ml/min. Fatty acid methyl ester standards (Nu-Chek-Prep, Elysian, MN) were used for peak identification. For a better identification of the peaks two standards were used: GLC #464 which contains 52 fatty acids and a custom preparation, GLC #704, which contains 10 fatty acids, methyl esters of stearate, oleate, linoleate, alpha linolenate, gamma linolenate, homogamma linolenate, arachidonate, eicosapentaenoate, docosapentaenoate, and docosahexaenoate. The fatty acid methyl esters were reported as the percent of the total methylated fatty acids (area under the curve). Using this protocol and column, we could not clearly separate the oleic acid (18:1n-9) and alpha linolenic acid (ALA 18:3n-3) peaks thus we report these results as 18:1 + 18:3 ALA. However, the differences in the peaks due to diet should be mostly ALA since it is the dietary ALA that was altered. A T-test was used to determine statistical differences of individual fatty acids between dietary groups.

### Gene expression assay

The Mouse Signal Transduction Pathway Finder™ RT^2 ^*Profiler*™ PCR Array, PAMM-014 (SuperArray Bioscience Corporation, Frederick, MD) was used to analyze the expression of genes in 3-4 mammary glands per group at 130 days of age mice. (The complete list of genes on the plate can be found at http://www.sabiosciences.com/rt_pcr_product/HTML/PAMM-014A.html.) Frozen tissue was homogenized in Tri Reagent (Sigma-Aldrich, St. Louis, Mo) following the protocol of the manufacturer to isolate the RNA. RNA quality control was performed for all samples to insure the purity and integrity of the RNA on an Agilent 2100 Bioanalyzer (Santa Clara, CA). The RT2 First Strand Kit was used to make cDNA; the cDNA was then quantitatively amplified by real time PCR using an ABI Prism 7000 (Applied Biosystems, Foster City, CA) and RT2 qPCR Master Mix (Superarray) according to the manufacturer's protocol. The protocol and software provided by SuperArray were followed to determine relative fold difference in gene expression using the ΔΔct method and for statistical analyses of the data by T-test.

### Immunoblot analysis

Frozen mammary gland tissues were homogenized in tissue extraction buffer (50 mM Tris, pH 7.4, 250 mM NaCl, 5 Mm EDTA, 2 mM Na_3_VO_4_, 1 mM NaF, 20 mM Na_4_P_2_O_7_, 0.02% NaN_3 _and proprietary detergent) from BioSource International, Inc. at 4°C to prepare cell lysates. Protein concentration was determined by BCA protein Assay Kit (EMD Biosciences, Inc. Darmstadt, Germany) following the manufacturer's protocol. Ten micrograms of protein were applied to each lane of a 4-15% Tris-HCl polyacrylamide gradient gel (Bio-Rad, Hercules, CA), separated by electrophoresis and then transferred onto a nitrocellulose membrane. The blots were blocked with 5% BSA in TBST overnight at 4°C and probed with primary antibodies against [CCAAT-enhancer binding proteins β (C/EBPβ) (Santa Cruz Biotechnology, Inc., Santa Cruz, CA), fatty acid synthase (Santa Cruz Biotechnology, Inc., Santa Cruz, CA), glyceraldehyde 3 phosphate dehydrogenase (GAPDH) (CHEMICON International, Billerica, MA) or cytokeratin 8 (Santa Cruz Biotechnology, Inc., Santa Cruz, CA,)] in blocking buffer for 1 hour at room temperature. The membrane was thereafter incubated with antimouse (Santa Cruz Biotechnology, Inc.) secondary antibody horseradish peroxidase (HRP) conjugate followed by signal detection with chemiluminescence (ECL Kit, PIERCE, Inc.). Densitometry was used to quantify bands. A ChemDoc XRS system (Bio-Rad Laboratories Inc., Hercules, CA) was used to acquire the image then image analysis was done using 'Quantity One' software, V. 4.5.2 (Bio-Rad Laboratories Inc., Hercules, CA). Data was normalized by cytokeratin (for size of the epithelial compartment) and by GAPDH (for protein loading).

## Results

### Group nomenclature

Groups will be referred to as: 1) CO/CO - mothers fed the 10% corn oil diet, pup weaned to the 10% corn oil diet or 2) CA/CO - mothers fed the 10% canola oil diet, pups weaned to the 10% corn oil diet. Pups were NOT exposed to the 10% canola oil diet after weaning.

### Body weight gain

**Figure 1:**
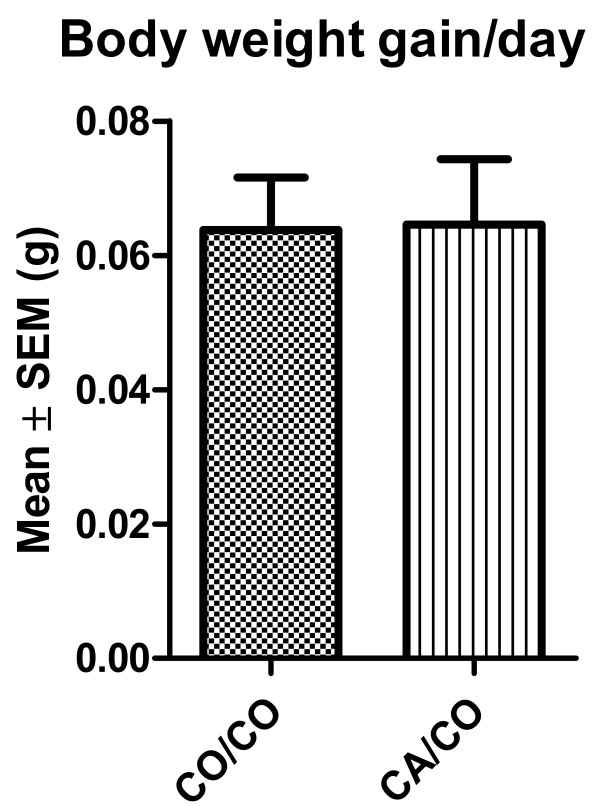
**Mean body weight gain between 21 and 130 days of age**. Body weight gain between 21 and 130 days of age was determined for each mouse. There was no significant difference between the two groups in body weight gain between weaning (21 days) and 130 days of age. CO/CO n = 14; CA/CO n = 17 mice, p = 0.95 by T-test.

Mice were allowed free access to food. There was no difference due to maternal diet in the amount of weight gained between weaning and 170 days of age by the groups of experimental mice (p = 0.95 by T-test). Figure 1 shows the mean amount of weight gained per mouse per day from weaning until 170 days.

### Diet influence on tissue lipid composition

The lipid compositions of the livers and mammary glands at 21 and 130 days of age are shown in Table 2.

**Table 2:** Major omega 6 and omega 3 fatty acids in tissues of mice.

Liver 21 days			
**Fatty acid**	**Corn oil diet**	**Canola diet**	**p value**

18:2 LA	17.35 ± 1.22	12.10 ± 0.90	<0.0001*

18:1+18:3 ALA	9.59 ± 2.16	20.29 ± 2.04	<0.0001*

20:4 AA	18.40 ± 1.60	10.08 ± 0.57	<0.0001*

20:5 EPA	0.09 ± 0.02	1.39 ± 0.20	<0.0001*

22:6 DHA	5.04 ± 0.81	11.22 ± 0.46	<0.0001*

22:5 DPA	0.64 ± 0.08	0.53 ± 0.06	0.023*

Liver 130 days			

18:2 LA	23.96 ± 2.74	22.91 ± 0.93	0.5565

18:1+18:3 ALA	18.19 ± 2.47	24.14 ± 1.01	0.0289*

20:4 AA	13.82 ± 0.46	10.33 ± 0.49	0.0041*

20:5 EPA	0.045 ± 0.04	0.10 ± 0.04	0.2382

22:6 DHA	5.37 ± 0.49	3.83 ± 0.17	0.0127*

22:5 DPA	0.315 ± 0.04	0.24 ± 0.03	0.0817

Mammary gland 21 days			

18:2 LA	26.25 ± 1.32	6.74 ± 1.99	0.0000*

18:1+18:3 ALA	29.26 ± 0.70	50.99 ± 1.98	0.0000*

20:4 AA	0.67 ± 0.12	0.31 ± 0.11	0.0043*

20:5 EPA	0.06 ± 0.01	0.08 ± 0.05	0.4050

22:6 DHA	0.08 ± 0.02	0.02 ± 0.01	0.0008*

22:5 DPA	0.01 ± 0.01	0.06 ± 0.03	0.0124*

Mammary gland 130 days			

18:2 LA	37.15 ± 0.78	35.77 ± 2.30	0.2879

18:1+18:3 ALA	0.40 ± 0.01	0.38 ± 0.03	0.1996

20:4 AA	0.13 ± 0.01	0.11 ± 0.03	0.3218

20:5 EPA	0.02 ± 0.01	0.00 ± 0.00	0.0000*

22:6 DHA	0.01 ± 0.00	0.01 ± 0.01	0.2415

22:5 DPA	0.02 ± 0.00	0.00 ± 0.00	0.0000*

*values in row are significantly different by T-test

The percents of assayed fatty acids of liver and mammary gland at 21 and 130 days old are shown. The results of T-tests between each pair of treatments for each tissue and time-point are shown, n = 3 to 5 mice per diet per time point. A p < 0.05 is taken to be statistically significant and is further indicated by an *

At 21 days of age (the time of weaning), both the livers and the mammary glands of pups from the CA mothers contained significantly less (p < 0.05 by T-test) of the n-6 PUFA (LA and AA) than the livers and mammary glands of pups from the CO mothers.

At 21 days of age, the livers of pups from the CA mothers contained significantly more (p < 0.05 by T-test) of the n-3 PUFA, ALA, and significantly more (p < 0.05 by T-test) of the products of ALA metabolism, EPA and DHA than the livers of pups from the CO mothers. The only source of the omega 3 bond was the ALA in the canola oil. Thus, the ALA in the canola oil was effectively elongated and desaturated to the longer chain omega 3 PUFAs, EPA and DHA, for incorporation into lipids in the liver.

At 21 days of age, the mammary glands of pups from the CA mothers contained significantly more (p < 0.05 by T-test) ALA and DPA than the pups from the CO mothers. The content of the longer chain fatty acids: AA, EPA, DHA and DPA in the mammary glands of both groups was less that 1% each indicating less incorporation of long chain fatty acids or less activity of elongation and desaturation enzymes in the mammary glands than in the liver.

All mice were fed the corn oil diet from 21 to 130 days of age. At 130 days of age the livers of the CA/CO group still contained significantly more (p < 0.05 by T-test) ALA (n-3 PUFA) and significantly less (p < 0.05 by T-test) AA (n-6 PUFA) than the CO/CO group. The LA, ALA, and AA in the mammary glands of the two groups were not different. EPA, DHA and DPA were almost undetectable in the mammary glands at 130 days of age, though the small differences between groups for EPA and DPA were statistically significant. Both EPA and DHA were significantly less (p < 0.05 by T-test) in the mammary glands of the CA/CO than in the CO/CO group.

### Effect of maternal consumption of canola oil instead of corn oil on tumor multiplicity, incidence and growth

The total number of tumors and the tumor weight per mouse were assessed in C3(1)T_AG_/129 female offspring at 110, 130, 150, 170 days of age. The presence of the transgene was confirmed in all pups used in the experiment (data not showed).

### Incidence

**Figure 2:**
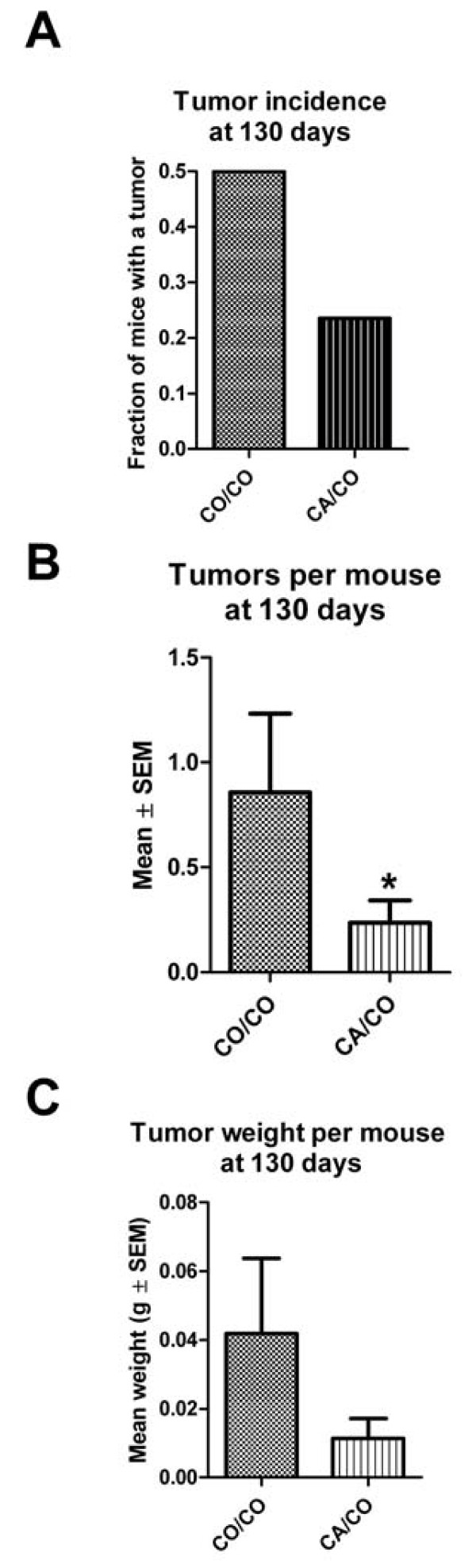
**Tumor incidence and multiplicity at 130 days of age**. A. Tumor incidence (fraction of mice with any tumor) at 130 days of age was not quite significantly different due to the diet of the mother. CO/CO -- 7 of 14 mice had tumors, CA/CO -- 4 of 17 mice had tumors. Fisher's exact test, p = 0.1. B. Tumor multiplicity, the mean number of tumors per mouse at 130 days of age. There were significantly fewer tumors per mouse by Mann-Whitney, p < 0.001. CO/CO n = 14 mice; CA/CO n = 17 mice. C. The total tumor weight per mouse was not quite significantly less in the CA/CO group than in the CO/CO group, p = 0.15 by Mann-Whitney test. CO/CO n = 14 mice; CA/CO n = 17 mice.

At 110 days of age, no mice had tumors. The tumor incidence (whether or not a mouse has a tumor) at 130 days of age is shown in Figure 2A. The tumor incidence of the CA/CO group, (4/17 or 23%) was not quite significantly less (p = 0.1) by Fisher's exact test than the tumor incidence of the CO/CO group, (7/14 or 50%). At 150 and 170 days of age, all mice had at least one tumor, thus the tumor incidence was 100%.

### Multiplicity

**Figure 3:**
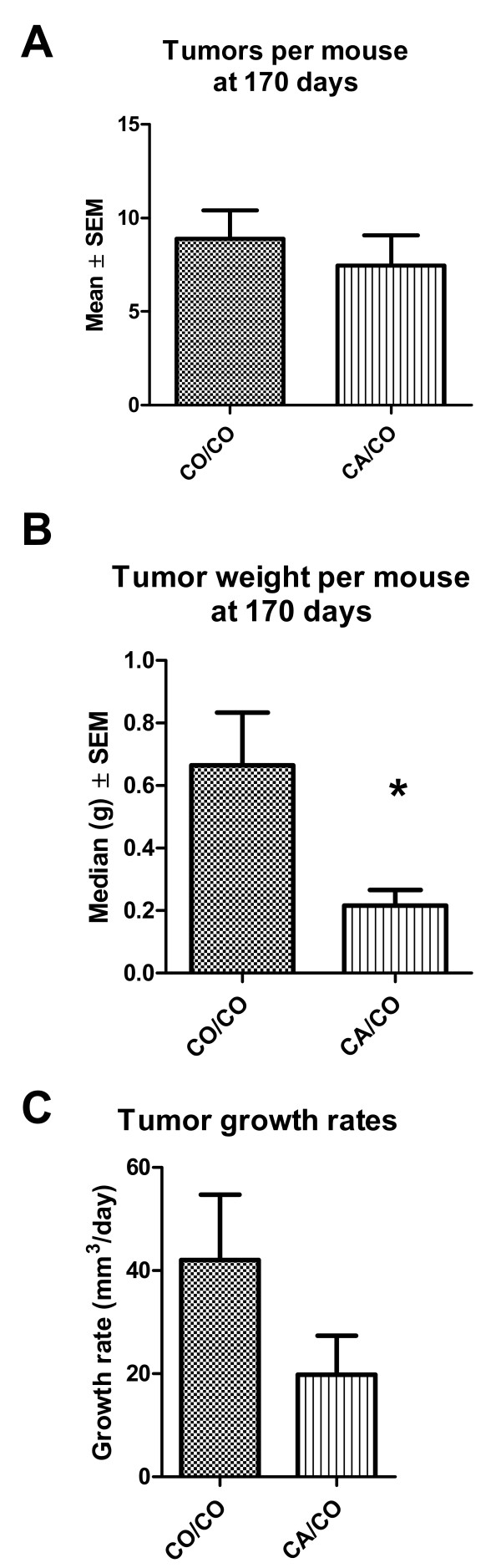
**Tumor multiplicity at 170 days and growth rate**. A. The mean number of tumors per mouse at 170 days was less, but not significantly different, in the CA/CO group (n = 11) than in the CO/CO group (n = 9). B. The tumor weight per mouse at 170 days was significantly less in the CA/CO group (n = 11) than in the CO/CO group (n = 9), p < 0.02 by Mann-Whitney. C. The mean tumor growth rate in the CA/CO group from detection until 170 days of age was 1/2 the growth rate of the CO/CO group.

Since these mice all bear a tumor promoting transgene, all mice are expected to develop tumors at some point. As shown in Figure 2B, the tumor multiplicity (number of tumors per mouse) at 130 days of age was significantly less (p < 0.001 by Mann-Whitney) in the CA/CO group than in the CO/CO group. Even at 170 days of age, Figure 3A, the multiplicity of tumors in the CA/CO group was slightly less than in the CO/CO group.

### Total tumor weight

The total tumor weight per mouse, calculated from autopsy data, indicates a difference in tumor burden due to the diet of the mother during gestation and lactation of the offspring. At 130 days of age, Figure 2-C, the tumor weight in the CA/CO group was not quite significantly less (p = 0.15 by Mann-Whitney) than that of the CO/CO group. By 170 days of age, Figure 3-B, the tumor weight per mouse (due to both fewer tumors/mouse and slower growth of tumors that developed) in the CA/CO group was significantly less (p = 0.02 by Mann-Whitney) than that of the CO/CO group.

### Tumor growth rate

The mean tumor growth rates, calculated from measured tumor growth of 11 tumors for the CO/CO group and 8 tumors for the CA/CO group, are shown in Figure 3C. Linear regression analyses were used to determine the growth rate of each tumor (data not shown). A T-test of the tumor growth rates showed that the mean tumor growth rates were slower in the CA/CO group but not quite significantly different, p = 0.11.

### Number of glands with tumor

**Figure 4:**
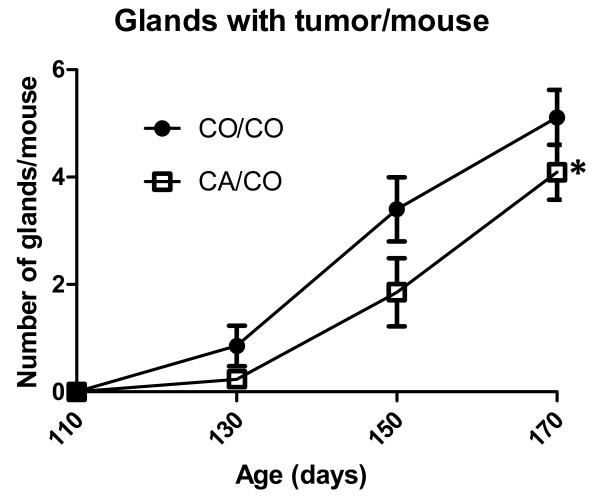
**Glands with tumor per mouse with time**. Two way analyses of variance showed that the number of glands with tumor per mouse with time was significantly different due to treatment (p = 0.02) and to time (p = 0.0001). Consumption of the canola oil containing diet by the mothers of these mice significantly decreased the number of glands with tumor. As expected, with time the number of glands with tumor increased in both groups. Number of mice per group at 110, 130, 150 and 170 days of age are: CO/CO - 14, 14, 5, 9, and CA/CO -- 6, 17, 7, 11 respectively.

The number of glands with tumor at each time point is illustrated in Figure 4. Two way analysis of variance revealed that there were significant effects due to both diet and time. The number of glands with tumor was significantly less in the CA/CO group than in the CO/CO group, p = 0.02, and as expected, the number of glands with tumor was significantly increased with time, p < 0.0001.

Taken together, these data indicate that maternal consumption of canola oil delayed appearance of tumors in these transgenic mice and slowed the growth rate of the tumors that arose, resulting in significantly reduced tumor burden at 170 days of age.

### Effect of maternal diet on gene and protein expression at 130 days of age

Analysis of expression of genes involved in multiple cell signaling pathways in mammary glands of mice at 130 days of age was used to identify potential mechanisms for how maternal consumption of canola oil might slow mammary gland tumorigenesis in C3(1)T_AG_/129 mice offspring. We chose this time point since at 130 days of age there were mammary glands without tumor in each group so that changes in gene expression due to the maternal diet but not those due to the presence of a tumor could be assessed. *A priori*, we did not know which signal transduction pathway(s) might be important for any alterations in tumor development. The Mouse Signal Transduction Pathway Finder™ reverse transcriptase, real time PCR (rtPCR) panel profiles the expression of 84 key genes representative of 18 different signal transduction pathways. The CO/CO group was the control group, the CA/CO group was the experimental group for analysis. The presence of the large T antigen protein in each mammary gland was confirmed by Western Blot (data not shown).

Table 3 presents genes that were analyzed and found to be at least 2 fold different between the two groups at 130 days of age. Differences in gene expression between groups at 130 days of age must be due to sustained gene expression changes induced by the maternal diet since: 1) both groups were weaned to the same diet 109 days previously and 2) there are only very small differences in fat composition in the mammary gland at 130 days of age and these differences do not support the notion that increased n-3 PUFA in the CA/CO group is influencing gene expression. The possible significances of some of the changes in gene expression are presented in the discussion.

**Table 3:** Genes expression in mammary glands at 130 days of age.

Gene name	Symbol	Fold change CA/CO vs CO/CO
Bcl2-associated X protein	Bax	22.71

B-cell leukemia/lymphoma 2	Bcl2	4.01

Bcl2-like 1	Bcl2l1	-9.92

Baculoviral IAP repeat-containing 3	Birc3	-2.35

Breast cancer 1	Brca1	-2.35

Chemokine (C-C motif) ligand 2	Ccl2	-2.25

Cyclin-dependent kinase inhibitor 2A	Cdkn2a	6.36

CCAAT/enhancer binding protein (C/EBP), beta	Cebpb	550.65

Chemokine (C-X-C motif) ligand 9	Cxcl9	2.02

Early growth response 1	Egr1	13.13

Etoposide induced 2.4 mRNA	Ei24	-2.15

Engrailed 1	En1	3.48

Fatty acid synthase	Fasn	-7.09

Fibronectin 1	Fn1	-4.42

Hedgehog-interacting protein	Hhip	-2.58

Hexokinase 2	Hk2	4.63

Homeo box A1	Hoxa1	-2.07

Intercellular adhesion molecule	Icam1	-2.05

Insulin-like growth factor binding protein 3	Igfbp3	-2.51

Inhibitor of kappaB kinase beta	Ikbkb	-3.13

Interleukin 1 alpha	Il1a	-2.06

Interleukin 2 receptor, alpha chain	Il2ra	-3.33

Interleukin 4 receptor, alpha	Il4ra	-2.19

Lymphoid enhancer binding factor 1	Lef1	-3.02

Lymphotoxin A	Lta	-3.19

Matrix metallopeptidase 10	Mmp10	2.87

Matrix metallopeptidase 7	Mmp7	2.97

Ngfi-A binding protein 2	Nab2	-4.71

Nitric oxide synthase 2, inducible, macrophage	Nos2	-3.31

Patched homolog 1	Ptch1	-3.32

Transcription factor 7, T-cell specific	Tcf7	2.53

Telomerase reverse transcriptase	Tert	-2.55

Transferrin receptor	Tfrc	-2.04

Transmembrane, prostate androgen induced RNA	Tmepai	-2.52

Tumor necrosis factor	Tnf	-5.27

Transformation related protein 53	Trp53	-2.74

Vascular cell adhesion molecule 1	Vcam1	-3.16

Wingless-related MMTV integration site 2	Wnt2	-2.24

Mice were exposed to maternal consumption of either corn or canola oil diets. Real time, reverse transcriptase PCR was performed to determine mRNA abundance in mammary glands of experimental mice at 130 days of age. Genes with greater than 2 fold differences in mRNA abundance, of the genes analyzed (see methods), are shown. To better differentiate differences, results of genes with increased expression in the CA/CO group are shifted to the left, results of genes with decreased expression in the CA/CO group are shifted to the right.

**Figure 5:**
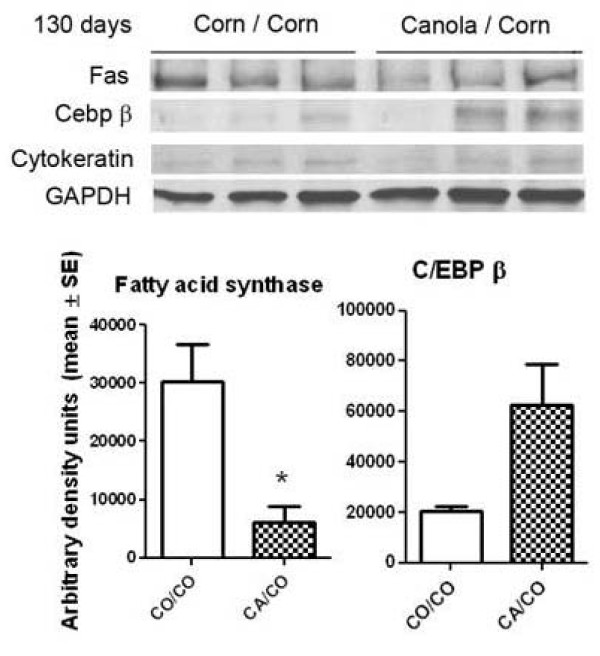
**Results of protein analyses for fatty acid synthase (Fas) and CCAAT/enhancer binding protein (C/EBP), beta at 130 days of age**. Western blot for fatty acid synthase showed that Fas protein was significantly less (p = 0.02 by T-test, n = 3/group) and that C/EBP, β was not quite significantly higher (p = 0.06 by T-Test, n = 3/group) in mammary glands of CA/CO mice than in CO/CO mice at 130 days. Values shown are relative density and have been corrected for protein loading (GAPDH) and for cytokeratin (to correct for the epithelial compartment of the assayed sample).

The differences in mRNA should be reflected in changes in protein abundance. Figure 5 illustrates the results of Western blot for two genes of interest, fatty acid synthase (Fas) and CCAAT/enhancer binding protein β (C/EBPβ). The mouse mammary gland contains a high percentage of adipocyte as well as the epithelial cells that form cancer, thus, the protein change was normalized by the size of the epithelial cell compartment in the specimen, using cytokeratin, and for protein loading using GAPDH. Compared to the CO/CO group, at 130 days of age, Fas protein was significantly less in the CA/CO group whereas C/EBPβ protein was increased in the CA/CO group.

## Discussion

The results of this study indicate that incorporation of canola oil in the maternal diet delayed the development of mammary gland cancer in this transgenic mouse model. The mice exposed to canola oil during gestation and lactation had significantly fewer tumors per mouse and the tumor incidence was 1/2 that of the control mice at 130 days of age. By 170 days of age the canola exposed mice had almost as many tumors as the control mice. However, since the growth rate of the tumors that developed in the canola oil exposed mice was about 1/2 that of the control mice and the appearance of tumors was delayed, the tumor burden (tumor weight) in the canola exposed mice at 170 days of age was significantly less than that of the control mice. If these results can be extrapolated to humans they are important for 2 reasons: 1) incorporation of canola oil in the diet is an easy dietary change for humans to make and 2) the maternal diet can have a life-long influence on development of breast cancer in the daughter.

Epidemiologic studies can be interpreted to support the notion that the maternal diet can influence breast cancer risk in the daughter. When Chinese, Japanese or Filipino women migrate to the United States, breast cancer rates rise over two generations to approach that of US women [27]. Breast cancer incidence in first generation migrants (who consumed a Western diet but whose mothers consumed the traditional diet) was increased almost 3 fold over that of the Asian-born mother, but was still lower than that of the general Western population, indicating that there remained some protection from breast cancer due to the mother's traditional diet. In second generation migrants, whose mothers consumed a Western diet during gestation and lactation of the daughter, breast cancer risk was 5-fold higher than the breast cancer risk for the Asian born grandmother [28] and was the same as the general Western population.

What could explain the benefit of exposure to maternal consumption of the omega 3 fatty acids in canola oil? The fatty acid composition of the mammary glands was different between the two groups at 21 days of age, but by 130 days of age there were no real differences in fatty acid composition. However, at 130 days of age, there were significant differences in gene expression in the mammary glands of these mice. The PCR array that we chose assays mRNA abundance in 84 key genes representative of 18 different signal transduction pathways that are important to the development of cancer. We found that there were multiple differences in gene expression between the 2 groups of mice when the mice were 130 days of age, 109 days after the last exposure to the diet that contained canola oil.

Among the differential changes that were of special interest was the large increase in CEBPβ(CCAAT/enhancer binding protein β) mRNA. Western blot confirmed that the mRNA was being translated to protein and that the quantity of protein was higher in the CA/CO group. CEBPβ is a leucine zipper transcription factor. The expression of CEBPβ in the liver has been shown to respond to dietary changes [29]. Homodimers and heterodimers of CEBPβ initiate transcription of multiple factors involved in proliferation, differentiation and apoptosis in the mammary gland [30]. Multiple isoforms of the protein, (including some that are dominant negatives) may be generated by truncation or proteolysis of the CEBPβ transcript [31]. Slowed proliferation and increased differentiation could result in reduced tumor incidence while promotion of apoptosis could slow tumor growth. These mechanisms are supported by the phenotypic data however the exact meaning of increased CEBPβ expression in the mammary gland in this model will require additional study.

The 7 to 8 fold decrease of fatty acid synthase mRNA and significant decrease in fatty acid synthase protein by maternal consumption of the canola oil diet is particularly interesting. Increased expression of fatty acid synthase has been associated with the early steps of human mammary carcinogenesis [32]. Conversely, inhibition of fatty acid synthase has been associated with apoptosis of human breast cancer cells [33] and has been suggested as a target for chemoprevention of breast cancer [34]. In cell culture studies, α-linolenic acid (increased in the canola oil diet) has been shown to be tumoricidal to breast cancer cells and to inhibit the overexpression of fatty acid synthase [35]. The long term suppression of fatty acid synthase would contribute to reduced tumor burden seen in this model.

Another gene expression change that could reduce tumor burden was the 13 fold increased expression of Egr1 mRNA in the CA/CO group. Egr1 is a tumor suppressor gene that has been associated with suppression of proliferation [36]. The expression of this gene has been shown to be increased by genistein and by retinoids [36,37] providing precedent for the regulation of this gene by dietary components.

Even though we did not directly assess NFκB activation, the mRNA results suggest that activation of the transcription factor NFκB was reduced in mammary glands of pups from mothers that consumed the canola oil diet. The mRNA for inhibitor of κB kinase β (IKKβ) was reduced 3 fold in CA/CO pups at 130 days of age. Activation of IKKβ results in the phosphorylation of IκB (inhibitor of κB) and allows formation of NFκB dimers that can translocate to the nucleus and activate transcription of downstream genes [38]. Activation of IKKβ is an important regulatory step in NFκB activity [38], thus reduced IKKβ would be expected to result in decreased activation of NFκB and reduction in mRNA of genes that are transcribed following NFκB binding. Genes downstream from NFκB include inducible nitric oxide synthase (iNOS), tumor necrosis factor (TNF) and vascular cell adhesion molecule 1 (Vcam1). The mRNA from each of these genes was decreased 2 to 5 fold, supporting the idea that NFκB activation was reduced in the CA/CO pups. Clearly there is much work to be done to verify this notion and to identify mechanisms.

The mRNA changes in the Bcl-2 apoptotic pathway were also intriguing. Progression to apoptosis is a balance between pro-apoptotic genes such as Bcl-2 associated X (BAX) and anti-apoptotic genes such as Bcl-2 and Bcl-2-like-1 [39]. At 130 days of age, the mammary glands of CA/CO mice had BAX mRNA at 22 fold and BCL-2-like 1 at -10 fold that of the CO/CO mice. Bcl2 was increased 4 fold but clearly the overall balance is to promotion of apoptosis of defective epithelial cells in the CA/CO mammary glands.

## Conclusions

Clearly consumption of canola oil by mothers of the experimental mice delayed mammary gland tumor development in this model. Our gene expression data have provided clues to mechanisms employed but identifying and verifying the mechanism(s) remains to be done. Since long term changes in the expression of multiple proteins, such as seen in this study, are often related to epigenetic modification of the promoter region of genes, we have initiated epigenetic studies to identify these changes. Future work includes verification of protein changes and developing reasonable pathways for the delay in cancer development seen in this model.

It has been suggested that 30% or more of cancers could be prevented by dietary changes [40]. Substituting canola oil for corn oil would increase the ratio of omega 3 fatty acids in the diet and is an easy, cost effective dietary change for people to make. Many animal studies have shown that increasing omega 3 fatty acids in the adult diet provides multiple benefits against cancer. This work suggests that substituting canola oil for the corn oil in the maternal diet may decrease risk for breast cancer in the daughter in addition to providing benefit for the mother.

## Abbreviations

AA: arachidonic acid, a 20 carbon, n-6 PUFA with 4 unsaturations; ALA: alpha linolenic acid, an 18 carbon, n-3 PUFA with 3 unsaturations C3(1) SV40; TAg mouse: bears a transgene containing the 5' flanking region of the rat C3(1) prostate steroid binding protein to drive expression of the SV 40 large T-antigen; CA: canola oil containing diet; CA/CO: mother consumed canola oil containing diet, baby consumed corn oil containing diet after weaning, CEBPβ-CCAAT/enhancer binding protein β; CO: corn oil containing diet; CO/CO: mother consumed corn oil containing diet, baby consumed corn oil containing diet after weaning; DHA: docosahexaenoic acid, a 22 carbon, n-3 PUFA with 6 unsaturations; DPA: docosapentaenoic acid, a 22 carbon, n-3 PUFA with 5 unsaturations; EPA: eicosapentaenoic acid, a 20 carbon, n-3 PUFA with 5 unsaturations; Fas: fatty acid synthase; GAPDH: glyceraldehyde 3 phosphate dehydrogenase; IKKβ-inhibitor of κB kinase β; LA: linoleic acid, an 18 carbon, n-6 PUFA with 2 unsaturations; n-3: omega 3 fatty acid; n-6: omega 6 fatty acid; PUFA: polyunsaturated fatty acid

## Competing interests

The authors declare that they have no competing interests.

## Authors' contributions

WEH conceived the study, obtained funding, analyzed data and wrote the manuscript. GI performed the array studies and statistical analyses for the arrays, managed animal colonies. JAA assisted with all facets of the studies, performed protein analyses. All authors read and approved the final manuscript.

## Pre-publication history

The pre-publication history for this paper can be accessed here:

http://www.biomedcentral.com/1471-2407/10/81/prepub
